# Impact of Centralizing Gastric Cancer Surgery on Treatment, Morbidity, and Mortality

**DOI:** 10.1007/s11605-017-3531-x

**Published:** 2017-08-16

**Authors:** S. D. Nelen, L. Heuthorst, R. H. A. Verhoeven, F. Polat, Ph. M. Kruyt, K. Reijnders, F. T. J. Ferenschild, J. J. Bonenkamp, J. E. Rutter, J. H. W. de Wilt, E. J. Spillenaar Bilgen

**Affiliations:** 1 Department of Surgery, Radboud University Medical Center, Geert Grooteplein 10, Route 618, P.O. 9101, 6500 HB Nijmegen, the Netherlands; 2 Department of Research, Netherlands Comprehensive Cancer Organization (IKNL), Utrecht, the Netherlands; 3 Department of Surgery, Canisius-Wilhelmina Hospital, Nijmegen, the Netherlands; 4 Department of Surgery, Gelderse Vallei Hospital, Ede, the Netherlands; 5 Department of Surgery, Slingeland Hospital, Doetinchem, the Netherlands; 6 Department of Surgery, Maasziekenhuis Pantein, Boxmeer, the Netherlands; 7 Department of Surgery, Rijnstate Hospital, Arnhem, the Netherlands

**Keywords:** Stomach neoplasm, surgical procedures, Operative hospitals, Low-volume hospitals, High-volume

## Abstract

**Introduction:**

Centralization of gastric cancer surgery is thought to improve outcome and has been imposed in the Netherlands since 2012. This study analyzes the effect of centralization in terms of treatment outcome and survival in the Eastern part of the Netherlands.

**Methods:**

All gastric cancer patients without distant metastases who underwent a gastrectomy in six hospitals in the Eastern part of the Netherlands between 2008 and 2011 (pre-centralization) and 2013–2016 (post-centralization) were selected from the Netherlands Cancer Registry. Patient and tumor characteristics and treatment outcomes (duration of surgery, blood loss, resection margin, lymphadenectomy, chemotherapy, postoperative complications and hospital stay, and overall and disease-free survival) were analyzed and compared between pre- and post-centralization.

**Results:**

One hundred forty-four patients were included pre-centralization and 106 patients post-centralization. Patient and tumor characteristics were almost similar in the two periods. After centralization, more patients were treated with perioperative chemotherapy (25 vs. 42% *p* < 0.01). The proportion of patients treated with an adequate lymphadenectomy (21 vs. 93% *p* < 0.01*)* and laparoscopic surgery (6 vs. 40% *p* < 0.01) increased significantly (*p* < 0.01). The amount of cardiac complications (16 vs. 7.5% *p* < 0.05) decreased; however, complications needing a re-intervention were comparable (42 vs. 40% *p* = 0.79). Median hospital stay decreased from 10 to 8 days (*p* < 0.01). A 30-day mortality did not differ significantly (4.2 vs. 1.9%). A 1-year overall (78 vs. 80% *p =* 0.17) and disease-free survival (73 vs. 74% *p* = 0.66) remained stable.

**Discussion:**

Centralizing gastric cancer treatment in the Eastern part of the Netherlands resulted in improved lymph node harvesting and a successful introduction of laparoscopic gastrectomies. Centralization has not translated into improved mortality, and other variables may also have led to these improved outcomes. Further research using a nationwide population-based study will be needed to confirm these data.

## Introduction

Centralization of gastric cancer treatment is believed to improve outcome. Performing more gastrectomies per year in one center should lead to better surgical training and more specialized oncological (perioperative) care. A study from Denmark has shown improved outcomes of gastric cancer after centralization of gastric cancer surgery, including less anastomotic leakages, a decreased 30-day mortality, and an improved lymph node harvesting.^1^ In the Netherlands, centralization of gastric cancer surgery has been imposed in 2012 by mandating a minimum of ten gastrectomies per hospital per year and as of 2013, to a minimum of 20 per year. In the Netherlands, the benefit of centralization of gastric cancer treatment has not yet been proven.

Previous Dutch studies that were conducted have researched the influence of surgical training and hospital volume on survival in gastric cancer.^2^^,^ 3 Data from the West of the Netherlands have already showed in 2009 that after standardization and surgical training, relative 5-year survival rates increased for resected gastric cancer patients (41 vs. 52% *p* = 0.06).^2^ Other data from the Netherlands Cancer Registry (NCR) showed that high hospital volume was associated with a higher amount of harvested lymph nodes during surgery and pathology, but a difference in survival after gastrectomy was not demonstrated. This was probably caused by the limited amount of gastric cancer patients that were treated before 2009 in high volume hospitals (i.e., ≥ 20 patients per year).^3^

The aim of the present study was to examine the effects of centralization in terms of morbidity and mortality by comparing a cohort of surgical gastric cancer patients before (2008–2011) and after (2013–2016) centralization in the Eastern part of the Netherlands.

## Methods

Primary data were obtained from the population-based Netherlands Cancer Registry (NCR). This registry serves the total Dutch population of almost 17 million inhabitants. The NCR is based on notification of all newly diagnosed malignancies in the Netherlands by the national automated pathological archive (PALGA). Additional sources are the national registry of hospital discharge and radiotherapy institutions. Specially trained data managers of the NCR routinely extract information on diagnosis, staging, and treatment from the medical records. The information on vital status is obtained by an annual linkage with the Municipal Administrative Databases, which register all deceased and emigrated persons in the Netherlands. Tumor staging was performed according to the 6th and 7th UICC TNM classification. In order to reduce the influence of differences between the different TNM versions, the 7th UICC TNM classification was recoded into the 6th UICC TNM classification. Due to the fact that the 7th version of the UICC TNM classification is more specific than the 6th version, it was impossible to recode the 6th version into the 7th version of the UICC TNM classification. Tumor site within the stomach was coded based on the International Classification of Diseases for Oncology: proximal/middle (cardia, fundus, corpus, and lesser and greater curvature (C16.0, C16.1, C16.2, C16.5, C16.6)), pyloric and antrum (C16.3, C16.4), and overlapping or not otherwise specified (C16.8, C16.9).^4^ Tumor histology was coded according the Lauren classification.^5^

Additional data on comorbidity and complications were retrospectively registered by a data manager. Patient history, comorbidities, and American Society of Anesthesiologists (ASA)-classification were extracted from the preoperative anesthesiology report. Seven different comorbidities were registered: diabetes mellitus, immune compromising diseases, pulmonary disease, kidney failure, liver failure, cardiovascular disease, and/or gastrointestinal disease. Time between diagnosis and treatment was defined as the time between the diagnostic biopsy result and the first treatment (neoadjuvant chemotherapy or surgery).

The occurrence of postoperative complications and reinterventions were extracted from medical records. Complications were defined as any unwanted effect of primary treatment leading to reintervention within 30 days after surgery. Postoperative complications consist of anastomotic leakage, intra-abdominal abscess, wound infection, postoperative bleeding, pneumonia, urinary tract infection, and cardiac complications and were ranked according to the Clavien-Dindo classification.^6^ Postoperative reinterventions were defined as surgical-, radiological, endoscopic measures or antibiotic therapy within 30 days after surgery. Tumor recurrence was registered when patients had histologically proven recurrent gastric cancer or a strong suggestion on computed tomography and/or gastroscopy.

Before 2012, gastric cancer surgery in the Eastern part of the Netherlands was performed in six hospitals by 16 gastrointestinal surgeons of which only one surgeon performed laparoscopic surgery. After 2012, the gastric cancer surgery was centralized into one hospital (Rijnstate hospital) and was done by two teams with two gastrointestinal surgeons each. These four surgeons all performed laparoscopic gastrectomy. 2012 was a transitional year in which centralization was partly adopted and was therefore excluded from further analysis. For this study, we selected all patients with gastric cancer without distant metastases operated in the Eastern part of the Netherlands between January 2008 and December 2011 (pre-centralization) and patients who underwent gastrectomy in the Rijnstate hospital between January 2013 and June 2016 (post-centralization). In both pre- and post-centralization period, surgeons were well trained in laparoscopic surgery, and in the entire study period all patients were discussed in a multidisciplinary team meeting.

### Statistical Analysis

Descriptive statistics were used to characterize the patients before and after centralization; significance was calculated by means of chi-square or Mann-Whitney test. Treatment modalities and outcome of treatment in terms of postoperative complications and hospital stay were compared by chi-square or Mann-Whitney test.

Survival time was defined as time from surgery to death or until January 1, 2016 for patients who were still alive. With exception for patients who underwent surgery in 2016, for these patients, survival time was defined as time from surgery to death or until the last hospital visit registered in the medical file in December 2016. Disease-free survival was defined as time from surgery to tumor recurrence (regardless of the location of recurrence) or until the last hospital visit registered in the medical file in December 2016 in which there was no evidence on tumor recurrence. Kaplan-Meier curves were generated to examine the overall- and disease-free survival and compared by log-rank test. Multivariable Cox regression analyses were performed to investigate the prognostic impact of centralization on overall survival after adjustment for patient and tumor characteristics. To prevent over fitting due to limited amount of events in our study population, multivariable analysis was limited to five variables. Results from survival analyses using Cox regression analyses were reported as hazard ratios (HR) and 95% confidence interval (CI). Reported *p* values of < 0.050 were considered statistically significant. All analyses were conducted using IBM SPSS Statistics Version 23 (International Business Machines Statistical Package for the Social Sciences).

## Results

A total of 250 gastric cancer patients without distant metastasis who underwent gastric surgery were included. Before centralization, 144 patients (median follow-up time 43 months) were treated and 106 patients were treated post-centralization (median follow-up time 15 months, *p* < 0.01). Apart from variation in ASA-classification (*p <* 0.01), all other patient and tumor characteristics were comparable between both study populations (Table 1).

**Table 1:** Patient and tumor characteristics according to centralization status: pre-centralization 2008–2011 and post-centralization 2013–2016

		Pre-centralization (*N* = 144)	Percent	Post-centralization (*N* = 106)	Percent	Significance
Follow-up (months)		Median 43 IQR 48 (15–63)		Median 15 IQR 17 (7–24)		< 0.01*
Age in years	< 70	65	45	47	44	0.90^a^
	≥ 70	79	55	59	56	
Gender	Male	90	63	60	57	0.35^a^
	Female	54	38	46	43	
BMI		Mean 25.1 (SD 4.0)		Mean 25.4 (SD 4.0)		0.27*
ASA-classification	I	17	12	7	6.6	<0.02^a^
	II	81	56	74	70	
	III	35	24	21	20	
	IV	2	1.4	4	3.8	
	Unknown	9	6.3	0	0	
Comorbidity	0	44	31	31	29	0.79^a^
	1	63	44	45	43	
	≥ 2	36	25	30	28	
	Unknown	1	0.7	0	0	
Previous abdominal surgery	Yes	54	38	33	31	0.38^a^
	No	89	62	73	69	
	Unknown	1	0.7	0	0	
	Yes	12	8.3	6	5.7	0.49^a^
	No	131	91	100	94	
	Unknown	1	0.7	0	0	
Previous malignancy	Yes	20	14	20	19	0.40^a^
	No	123	85	86	81	
	Unknown	1	0.7	0	0	
pT-stage	0/×	10	6.9	9	9	0.36^a^
	1	29	20	23	22	
	2A	17	12	20	19	
	2B	55	38	38	36	
	3	27	19	15	14	
	4	6	4.2	1	0.9	
pN-stage	Nx	9	6.3	2	1.9	0.11^a^
	0	78	54	51	48	
	1	43	30	34	32	
	2	13	9	15	14	
	3	1	0.7	4	3.8	
Tumor topography	Proximal/middle	43	30	33	31	0.30^a^
	Pyloric/antrum	62	43	53	50	
	Overlapping/not specified	39	27	20	19	
Tumor grade	Well differentiated	8	5.6	4	3.8	0.12^a^
	Moderately differentiated	16	11	22	21	
	Poorly/undifferentiated	68	47	39	37	
	Unknown	52	36	41	39	
Type (Lauren classification)	Intestinal type	92	64	52	49	0.05^a^
	Diffuse type	45	31	49	46	
	Indeterminate type	7	4.9	5	4.7	

*Mann-Whitney test

^a^Pearson chi square

### Treatment

Centralization did not significantly affect time between diagnosis and start of treatment (Table 2). The amount of partial gastrectomies increased non-significantly, 68% between 2008 and 2011 and 74% between 2013 and 2016 (*p* = 0.34), and there was no significant increase in the number of microscopically radical resections (R0), 79% before centralization, and 87% after centralization (*p =* 0.16) (Table 2).

**Table 2:** Surgery and hospital stay according to centralization status: pre-centralization 2008–2011 and post-centralization 2013–2016

		Pre-centralization *N* = 144		Post-centralization *N* = 106		
			Percent		Percent	Significance
Time between diagnosis and treatment (days)		Median 35.50 IQR 23 (27–50)		Median 34.00 IQR 17 (28–45)		0.94*
Type of chemotherapy	None	68	47	47	44	0.65^a^
	Only neoadjuvant	37	26	14	13	0.02^a^
	Only adjuvant	3	2.1	1	0.9	0.44^a^
	Peri-operative	36	25	44	42	<0.01^a^
Type of resection	Partial gastrectomy	98	68	78	74	0.34^a^
	Total gastrectomy	46	32	28	26	
Resection method	Laparotomy	133	92	62	59	<0.01^a^
	Laparoscopic	8	5.6	42	40	
	Unknown	3	2.1	2	1.9	
Duration surgery (minutes)		Median 147 IQR 69 (116–185)		Median 180 IQR 74 (154–228)		<0.01*
Blood loss (ml)		Median 300 IQR 488 (200–688)		Median 200 IQR 313 (100–413)		<0.01*
Lymphnodes harvested	< 15	114	79	8	8	<0.01^a^
	≥ 15	30	21	98	93	
Tumor residue	R0	115	79	92	87	0.16^a^
	R1	22	15	13	12	
	Unknown	7	4.9	1	0.9	
Hospital stay (days)		Median 10 IQR 7 (7–14)		Median 8 IQR 4 (7–11)		<0.01*

*Mann-Whitney test

^a^Pearson chi square

After centralization, there was a significant increase in the use of perioperative chemotherapy (25 vs. 42%, *p* < 0.01); patients receiving no chemotherapy remained almost equal (47 vs. 44% *p* = 0.65), and the amount of patients receiving only neo-adjuvant treatment decreased significantly (26 vs. 13% *p* = 0.02). More patients were treated laparoscopically; respectively, 5.6 and 40% of all patients received a laparoscopic gastrectomy before and after centralization (*p* < 0.01). Median duration of surgery increased (147 vs. 180 min (*p* < 0.01), and the median amount of peroperative blood loss decreased (300 vs. 200 ml (*p* < 0.01). There was a significant increase of patients who had an adequate amount of more than 15 lymph nodes harvested during surgery from 21 towards 93% after centralization (*p* < 0.01). The median hospital stay decreased with 2 days after centralization (10 vs. 8 days *p* < 0.01) (Table 2.)

### Complications

No significant difference was seen in the amount and grade of postoperative complications (Table 3). The amount of anastomotic leakages did not decrease significantly, 9.0% between 2008 and 2011 and 6.6% between 2012 and 2016 (*p* = 0.49). The occurrence of an intra-abdominal abscess did not decrease after centralization (2.8 vs. 6.6% *p* = 0.15). However, the amount of patients with cardiac complications (16 vs. 7.5% *p* < 0.05) decreased significantly after centralization (Table 4).

**Table 3:** Postoperative complications according the Clavien-Dindo classification of surgical complications

	Pre-centralization (*N* = 144)		Post-centralization (*N* = 106)		*p* value
	Number	Percent	Number	Percent	
< II	84	58	65	61	0.51
II	32	22	23	22	
III	10	7	11	10	
IV	11	8	5	5	
V	7	5	2	2

**Table 4:** Postoperative complications in 30 days after surgery according to centralization status: pre-centralization 2008–2011 and post-centralization 2013–2016

	Pre-centralization (*N* = 144)		Post-centralization (*N* = 106)		*p* value
	Number	Percent	Number	Percent	
Postoperative complications	60	42	42	40	0.75
Anastomotic leakage	13	9.0	7	6.6	0.49
Intra-abdominal abscess	4	2.8	7	6.6	0.15
Wound infection	5	3.5	2	1.9	0.45
Postoperative bleeding	6	4.2	3	2.8	0.58
Pneumonia	29	20	19	18	0.66
Urinary tract infection	5	3.5	0	0	0.05
Cardiac complications	23	16	8	7.5	< 0.05

Additional analysis on the influence of type of surgery (i.e., laparoscopy vs. laparotomy) showed no significant difference in the amount of postoperative morbidity (39 vs. 42% *p* = 0.76).

There were no statistically significant differences in the amount of re-interventions between both study populations (Table 5). In both study periods, 12% of all patients underwent a postoperative surgical re-intervention. This was mainly caused by (a suspicion of) a failure of the gastrojejunostomy (pre-centralization 6.9% and post-centralization 6.6%). A 30-day mortality was 4.2 and 1.9%, respectively, (*p* = 0.17) before and after centralization. No statistically significant difference was seen in the 30-day mortality for both types of surgery (i.e., laparoscopy vs. laparotomy (2.0 vs. 3.6% *p* = 0.56).

**Table 5:** Postoperative re-interventions in 30 days after surgery according to centralization status: pre-centralization 2008–2011 and post-centralization 2013–2016

	Pre-centralization (*N* = 144)		Post-centralization (*N* = 106)		*p* value
	Number	Percent	Number	Percent	
Surgical intervention	17	12	13	12	0.91
Radiological intervention	11	7.6	3	2.8	0.10
Endoscopic intervention	2	1.4	3	2.8	0.42
Antibiotic therapy	48	33	36	34	0.92

### Recurrence and Survival

**Fig. 1:**
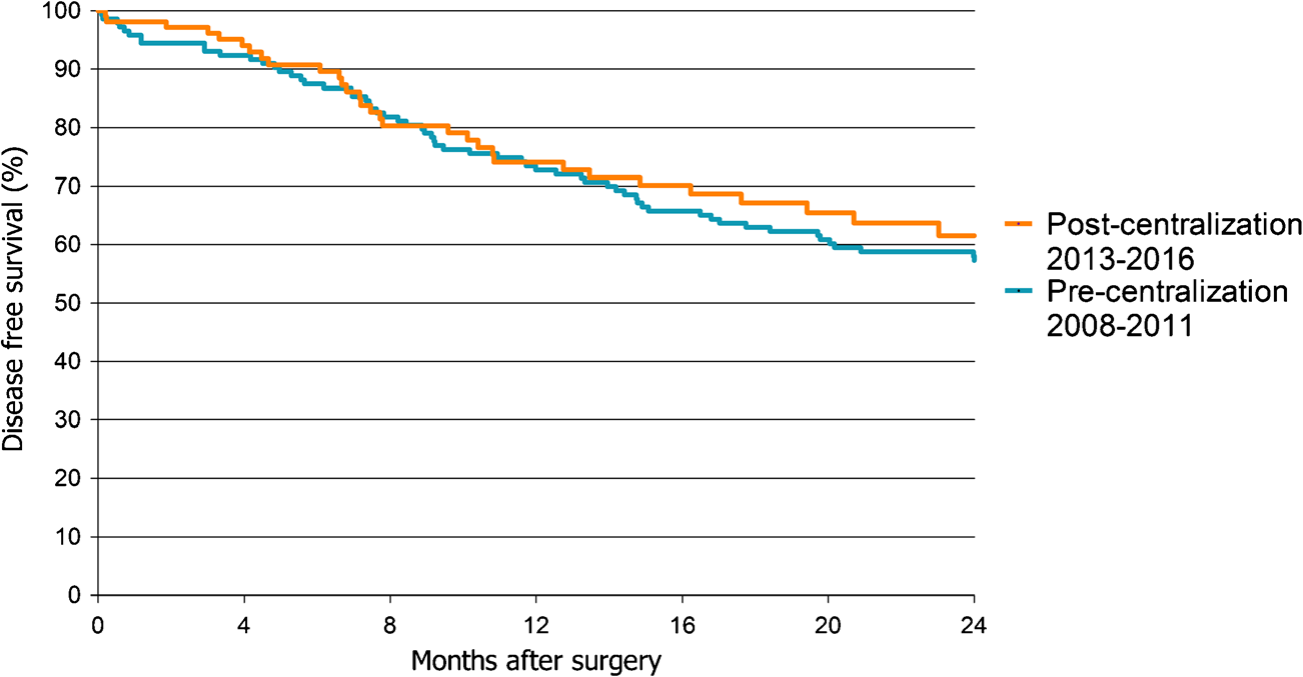
Kaplan-Meier 2-year disease-free survival curve. Patients grouped by centralization status. *p* = 0.66 (Log Rank test)

**Fig. 2:**
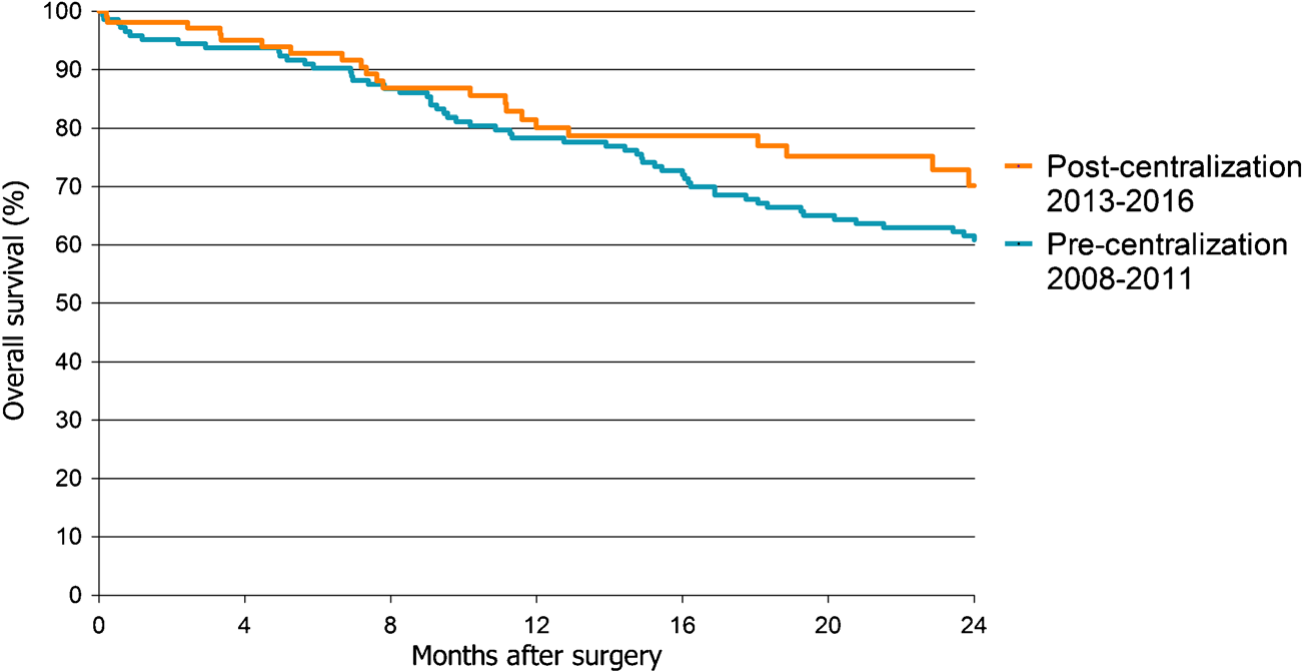
Kaplan-Meier 2-year overall survival curve. Patients grouped by centralization status. *p* = 0.17 (Log Rank test)

There were no statistically significant differences in disease-free and overall survival between patients treated before and after centralization in univariable and multivariable analysis. Kaplan-Meier survival analyses showed that a 1-year disease-free survival was 73% before and 74% after centralization (Fig. 1, *p* = 0.66). A 1-year overall survival of all patients treated before centralization was 78% and after centralization 80%; A 2-year overall survival was 62 versus 70% (Fig. 2, *p* = 0.17). Additional Kaplan-Meier survival analysis to examine the 2-year overall survival for both types of surgery (i.e., laparoscopy vs. laparotomy), showed no significant difference (Figure not shown, *p* = 0.35). Multivariable cox regression analysis showed no significant effect of centralization on overall survival (Table 6, HR = 0.6 95% C.I. 0.4–1.1 *p* = 0.08). However, an impact was seen of tumor stage on overall survival (Table 6, HR = 9.6 95% C.I. 4.6–19.7 *p* < 0.01).

**Table 6:** Cox regression analyses. The influence of different clinicopathological factors on survival

		Hazard ratio	95% CI	Significance
Centralization status	Post-centralization (Reference*:* Pre*-*centralization)	0.6	0.4–1.1	0.08
Age	≥ 70 (Reference *<* 70)	1.8	1.1–2.8	0.02
Comorbidity	0 (Reference)			
	1	1.0	0.6–1.6	1.0
	≥ 2	1.2	0.7–2.1	0.51
	Unknown	1.7	0.2–13.8	0.62
Tumor stage	Stage 1 (Reference)			
	Stages 2 and 3	2.8	1.8–4.3	< 0.01
	Stage 4	9.6	4.6–19.7	< 0.01
	Unknown	1.5	0.7–3.1	0.26
Chemotherapy	Yes (Reference: No)	0.8	0.5–1.3	0.42

## Discussion

This is the first study showing improved treatment outcome after centralization of gastric cancer treatment in the Netherlands since centralization had been effectuated in 2012. Gastric cancer patients significantly more often received perioperative chemotherapy and laparoscopic gastrectomy; the amount of perioperative blood loss significantly decreased and more patients received an adequate lymphadenectomy. After surgery, patients remained hospitalized for a significantly shorter period of time. Also after centralization, patients had significantly less postoperative cardiac complications, which might be caused by improved preoperative optimalization and perioperative monitoring.^3^^,^ 7 On the other hand, 30-day mortality, disease-free and overall survival did not improve significantly after centralization of gastric cancer treatment in the Eastern part of the Netherlands.

The introduction of laparoscopic gastrectomy in this region of the Netherlands, makes this study more difficult to interpret. It is suggested that laparoscopic gastrectomy causes less perioperative blood loss, fewer postoperative complications, shorter hospital stay, but equal surgical oncological results (i.e., tumor residue, lymph nodes harvest), and postoperative mortality.^8^^–^ 10 The abovementioned improved results of gastric cancer treatment after centralization could thus also be due to the introduction of laparoscopic surgery. However, introduction and performance of laparoscopic gastrectomy requires a certain number of resection per surgeon per time period. Therefore, it is safer and easier to perform laparoscopic gastric surgery in one high volume hospital than in six low volume centers. Various studies suggest that the learning curve of a laparoscopic gastrectomy is considered complete after more than 50 gastrectomies.^11^^,^ 12 This study showed that in our region, less than 40 gastrectomies are being performed per year. Dividing these cases over several hospitals, would have extended the surgeons learning curve for laparoscopic gastrectomy over several years.

The importance of centralizing low frequent and complex cancer care into high volume hospitals is endorsed globally^1^^,^ 3 ^,^ 13 ^–^ 15. Nevertheless, for gastric cancer, the benefit of centralizing treatment has not been proven beyond doubt; studies on centralization of gastric cancer care are often heterogeneous and occasionally conflicting.^16^^–^ 19 In recent years, several studies on centralization of gastric cancer care in the UK have been published.^17^^–^ 19 In those studies, both esophagus and gastric cancer are combined despite well-known differences in treatment, survival, and impact of centralization.^20^ Chan et al. reported decreased morbidity, mortality, and length of hospital stay after centralization.^17^ Next to that, the rate of patients treated with curative intent increased from 21 to 36%. However, another UK study in which survival was shown separately for gastric and esophagus cancer, showed no significant improvement of median survival after centralization for gastric cancer.^18^ Recently, a study by Mamidanna et al. demonstrated that mortality after gastrectomy was lower for surgeons with higher volumes.^15^ This study, done in over 12,000 patients showed that for each additional gastrectomy per year, 30-day mortality decreased significantly.^15^

After centralization of gastric cancer in the Eastern part of the Netherlands, there was a decrease in the incidence of surgically treated gastric cancer patients (*N* = 144 patients vs. *N* = 106 patients). Apart from the difference in the length of both study periods, this could be caused by the decreasing incidence of curative treatable gastric cancer.^21^ Furthermore, it could be possible that centralized hospitals perform a more critical preoperative selection, causing more patients to be excluded from surgical intervention.

Also an increased use of perioperative chemotherapy was shown in this study. One possible explanation for the increased use perioperative chemotherapy could be that centralization has led to stricter compliance of the Dutch guidelines for gastric cancer treatment which is introduced in May 2009. This guideline recommends perioperative chemotherapy with an epirubicin, cisplatin and/or fluorouracil based regime in patients with stage II and III gastric cancer*.* The guidelines were recently further expanded but recommendations considering perioperative chemotherapy remained similar.^22^ Besides that, the increased hospital volume after centralization has probably led to more experience in treating (high-risk) patients with perioperative chemotherapy and thereby better awareness of the possibilities, risks, and benefits of perioperative chemotherapy.

In this study, the increased use of perioperative chemotherapy was mainly due to increased use of adjuvant chemotherapy. A possible explanation for this is that patients in the post-centralization group had a shorter and/or better recovery after surgery (i.e., decreased hospital stay, less blood loss, and less postoperative complications). Postoperative complications are one of the main reasons for not starting postoperative chemotherapy.^16^ Completion of perioperative chemotherapy is able to improve 5-year overall and disease-free survival with more than 10%.^23^^,^ 24 However, in this retrospective study, the increased use of perioperative chemotherapy post-centralization did not result into an increased survival benefit. This is in line with the suggestion that adjuvant chemotherapy offers limited additional survival benefit for patients with curatively resected gastric cancer^25^^,^ 26.

The increased amount of harvested lymph nodes might be the effect of more experienced and trained surgeons in performing an adequate lymphadenectomy, but also due to the diligence and effort put in these time-consuming examinations by pathology departments.^27^ An adequate lymphadenectomy is especially relevant in order to predict prognosis, by assessing an adequate and reliable UICC TNM N-stage.^28^ But evidence also suggests that resection of 15 or more lymph nodes resulted in an improvement of 10-year disease-specific survival with more than 15%.^29^^–^ 31 In the present study, a survival benefit due to the improved lymphadenectomy could not be demonstrated. This might be caused due to the suggestion that surgical lymphadenectomy before and after centralization remained equal, but harvesting of lymph nodes by pathology department improved after centralization. Variation in evaluated lymph nodes between pathology departments has been studied before in the Netherlands.^27^ Lemmens et al. investigated the median-evaluated lymph nodes in six different pathology departments between 1999 and 2007 and showed a variation between 5 and 9 median evaluated nodes.^27^ Another explanation for the lack of survival benefit after centralization might be the low number of patients.

This study showed no significant improvement in the 30-day mortality and the 1-year overall survival. Before centralization, 30-day mortality (4.2%) was already lower than the nationwide average of 6.9%.^32^ Mortality rates after gastric surgery in the Eastern part of the Netherlands were also among the lowest compared to various European countries, ranging from 3.5 to 6.9%^32^ and only slightly higher than in the Danish study after their national centralization (2.4%).^1^ This relatively good outcome would be difficult to improve significantly after centralization. Moreover, producing a statistically significant difference in overall survival before and after centralization will probably require a larger study population and a longer follow-up. Possibly with a lager study population, the 40% risk reduction in multivariate analysis of the current study could result in statistical significance. Nevertheless, a postoperative mortality after centralization of 1.8% is a very satisfying outcome.

To conclude, this is the first retrospective cohort study to show a positive effect of centralizing gastric cancer treatment in the Netherlands. More perioperative chemotherapy, more harvested lymph nodes during surgery and/or pathology, less peroperative blood loss, and less postoperative cardiac complications, have however not yet led to a significant improvement of overall or disease-free survival. This is probably due to the small study population. A nationwide population based study will be needed to show a significant improvement in overall survival.

## Abbreviations

NCR: Netherlands Cancer Registration
UICC: Union for International Cancer Control
TNM: Tumor, node, metastasis
CI: Confidence interval
HR: Hazard ratio
OS: Overall survival

## References

[1] Jensen LS, Nielsen H, Mortensen PB, Pilegaard HK, Johnsen SP (2010). Enforcing centralization for gastric cancer in Denmark. European journal of surgical oncology: the journal of the European Society of Surgical Oncology and the British Association of Surgical Oncology..

[2] Krijnen P, den Dulk M, Meershoek-Klein Kranenbarg E, Jansen-Landheer ML, van de Velde CJ (2009). Improved survival after resectable non-cardia gastric cancer in the Netherlands: the importance of surgical training and quality control. European journal of surgical oncology : the journal of the European Society of Surgical Oncology and the British Association of Surgical Oncology..

[3] Dikken JL, Dassen AE, Lemmens VE (2012). Effect of hospital volume on postoperative mortality and survival after oesophageal and gastric cancer surgery in the Netherlands between 1989 and 2009. European journal of cancer..

[4] AG F (2000). International classification of diseases for oncology: ICD-O.

[5] Lauren P (1965). The two histological main types of gastric carcinoma: diffuse and so-called intestinal-type carcinoma. An attempt at a histo-clinical classification. Acta pathologica et microbiologica Scandinavica..

[6] Dindo D, Demartines N, Clavien PA (2004). Classification of surgical complications: a new proposal with evaluation in a cohort of 6336 patients and results of a survey. Annals of surgery..

[7] Dikken JL, Wouters MW, Lemmens VE (2012). Influence of hospital type on outcomes after oesophageal and gastric cancer surgery. The British journal of surgery..

[8] Kodera Y, Fujiwara M, Ohashi N (2010). Laparoscopic surgery for gastric cancer: a collective review with meta-analysis of randomized trials. Journal of the American College of Surgeons..

[9] Haverkamp L, Weijs TJ, van der Sluis PC, van der Tweel I, Ruurda JP, van Hillegersberg R (2013). Laparoscopic total gastrectomy versus open total gastrectomy for cancer: a systematic review and meta-analysis. Surgical endoscopy..

[10] Memon MA, Khan S, Yunus RM, Barr R, Memon B (2008). Meta-analysis of laparoscopic and open distal gastrectomy for gastric carcinoma. Surgical endoscopy..

[11] Zhang X, Tanigawa N (2009). Learning curve of laparoscopic surgery for gastric cancer, a laparoscopic distal gastrectomy-based analysis. Surgical endoscopy..

[12] Song JH, Choi YY, An JY, Kim DW, Hyung WJ, Noh SH (2015). Short-term outcomes of laparoscopic total gastrectomy performed by a single surgeon experienced in open gastrectomy: review of initial experience. Journal of gastric cancer..

[13] Geraedts M, de Cruppe W, Blum K, Ohmann C (2008). Implementation and effects of Germany's minimum volume regulations: results of the accompanying research. Deutsches Arzteblatt international..

[14] Birkmeyer JD, Sun Y, Goldfaden A, Birkmeyer NJ, Stukel TA (2006). Volume and process of care in high-risk cancer surgery. Cancer.

[15] Mamidanna R, Ni Z, Anderson O (2016). Surgeon volume and cancer esophagectomy, gastrectomy, and pancreatectomy: a population-based study in England. Annals of surgery..

[16] Thompson AM, Rapson T, Gilbert FJ, Park KG (2007). Hospital volume does not influence long-term survival of patients undergoing surgery for oesophageal or gastric cancer. The British journal of surgery..

[17] Chan DS, Reid TD, White C (2013). Influence of a regional centralised upper gastrointestinal cancer service model on patient safety, quality of care and survival. Clin Oncol (R Coll Radiol)..

[18] Boddy AP, Williamson JM, Vipond MN (2012). The effect of centralisation on the outcomes of oesophagogastric surgery—a fifteen year audit. Int J Surg..

[19] Coupland VH, Lagergren J, Luchtenborg M (2013). Hospital volume, proportion resected and mortality from oesophageal and gastric cancer: a population-based study in England, 2004-2008. Gut..

[20] Dikken JL, Lemmens VE, Wouters MW (2012). Increased incidence and survival for oesophageal cancer but not for gastric cardia cancer in the Netherlands. European journal of cancer..

[21] Nelen SD, Verhoeven RH, Lemmens VE, de Wilt JH, Bosscha K. Increasing survival gap between young and elderly gastric cancer patients. Gastric cancer. 2017. doi: 10.1007/s10120-017-0708-7. 10.1007/s10120-017-0708-7 PMC5658460 28275933

[22] Dutch Clinical Practice Guidelines for Gastric Carcinoma 2016. http://www.oncoline.nl/index.php?pagina=/richtlijn/item/pagina.php&richtlijn_id=740. Website last visited: May 1st 2017.

[23] Cunningham D, Allum WH, Stenning SP (2006). Perioperative chemotherapy versus surgery alone for resectable gastroesophageal cancer. The New England journal of medicine.

[24] Reece-Smith AM, Saha S, Cunnell ML (2012). MAGIC in practice: experience of peri-operative ECF/X chemotherapy in gastro-esophageal adenocarcinomas. Journal of surgical oncology..

[25] Hermans J, Bonenkamp JJ, Boon MC (1993). Adjuvant therapy after curative resection for gastric cancer: meta-analysis of randomized trials. Journal of clinical oncology : official journal of the American Society of Clinical Oncology..

[26] Paoletti X, Oba K, Burzykowski T (2010). Benefit of adjuvant chemotherapy for resectable gastric cancer: a meta-analysis. Jama.

[27] Lemmens VE, Dassen AE, van der Wurff AA, Coebergh JW, Bosscha K (2011). Lymph node examination among patients with gastric cancer: variation between departments of pathology and prognostic impact of lymph node ratio. European journal of surgical oncology: the journal of the European Society of Surgical Oncology and the British Association of Surgical Oncology..

[28] Nelen SD, van Steenbergen LN, Dassen AE, van der Wurff AA, Lemmens VE, Bosscha K (2013). The lymph node ratio as a prognostic factor for gastric cancer. Acta Oncol..

[29] Gholami S, Janson L, Worhunsky DJ (2015). Number of lymph nodes removed and survival after gastric cancer resection: an analysis from the US Gastric Cancer Collaborative. Journal of the American College of Surgeons..

[30] Marubini E, Bozzetti F, Miceli R, Bonfanti G, Gennari L (2002). Lymphadenectomy in gastric cancer: prognostic role and therapeutic implications. European journal of surgical oncology : the journal of the European Society of Surgical Oncology and the British Association of Surgical Oncology..

[31] Volpe CM, Driscoll DL, Douglass HO (2000). Outcome of patients with proximal gastric cancer depends on extent of resection and number of resected lymph nodes. Annals of surgical oncology..

[32] Dikken JL, van Sandick JW, Allum WH (2013). Differences in outcomes of oesophageal and gastric cancer surgery across Europe. The British journal of surgery..

